# Laser-based pulse oximetry eliminates pigmentation effects on oxygen saturation measurements: A pilot study

**DOI:** 10.1371/journal.pone.0333109

**Published:** 2025-10-06

**Authors:** Jonas A. Pologe, Nathan K. You, Mark Blumstein, Kristine L. Snyder, William W. Hay

**Affiliations:** 1 Research and Development, Zynex Monitoring Solutions, Boulder, Colorado, United States of America; 2 Denver, Colorado, United States of America; Radiation Application Research School, NSTRI, IRAN, ISLAMIC REPUBLIC OF

## Abstract

**Background:**

Almost since its introduction, pulse oximetry has been plagued by inaccuracy associated with pigmentation, whether from fingernail polishes or melanin. The presence of melanin in the optical path of a pulse oximetry sensor has been shown to artifactually increase oxygen saturation measurements which can result, clinically, in occult hypoxemia and misdiagnoses.

**Methods:**

This report describes the theoretical basis for this inaccuracy and presents results from both a benchtop study and a clinical study testing the effects of pigmentation on conventional LED-based pulse oximetry compared to laser-based pulse oximetry. The clinical portion of this study was performed on 18 consenting participants, nine darkly pigmented and nine lightly pigmented, to assess the ability of laser-based pulse oximetry to eliminate this dangerous pigmentation bias. The clinical study directly compared oxygen saturation readings on laser-based pulse oximeters to readings performed on two different LED-based pulse oximeters. All measurements were compared to invasive reference laboratory measurements performed on arterial blood samples. We hypothesized that monochromatic light sources used in laser-based pulse oximetry would make this new technology insensitive to pigmentation bias.

**Results:**

The clinical portion of this study showed significantly greater (p < 0.001) measurement error (variance) for the two LED-based pulse oximeters (5.48 and 5.47) compared to laser-based pulse oximetry (3.54), when analyzed for all participants. The bias differences in oxygen saturation measurements by the LED-based pulse oximeters, when made on lightly versus darkly pigmented participants compared to invasive reference measurements, accounts for most of the increase in measurement error.

**Conclusions:**

By combining theoretical development, experimental benchtop testing, and a clinical study, this research explains and demonstrates that the wide spectral bandwidth of LEDs is the root cause of pigmentation bias in commercially available LED-based pulse oximetry and validates the ability of narrow-band laser-based pulse oximetry to eliminate this pigmentation bias.

## Introduction

A major clinical problem with current pulse oximeters is that skin pigments can bias pulse oximeter measurements toward falsely higher values, particularly in patients at lower oxygen saturation levels [1–6]. In clinical practice, this bias has led to false assumptions about adequate oxygenation in darkly pigmented individuals, leading to reductions in oxygen delivery to these patients when the actual need was for increased oxygenation. This has led to adverse outcomes in more darkly pigmented individuals due to incorrectly restricted, and thus insufficient, oxygen supply [7–10].

The adverse effects of pigments on photoplethysmographic (pulse oximetry) measurements of arterial oxygen saturation (the percentage of hemoglobin in the arterial blood that is bound to oxygen) have been documented at least since 1987 [1–3]. In humans undergoing pulse oximetry monitoring, these pigments have primarily included melanin in highly variable degrees depending on genetic backgrounds. Other pigments that can affect pulse oximetry readings include those in nail polish, as pulse oximeter probes commonly monitor through the nails of the fingers [11]. Published reports, however, have been conflicting as to the extent, and even the presence, of pigmentation bias in any given type of pulse oximeter [12–14].

**Fig 1:**
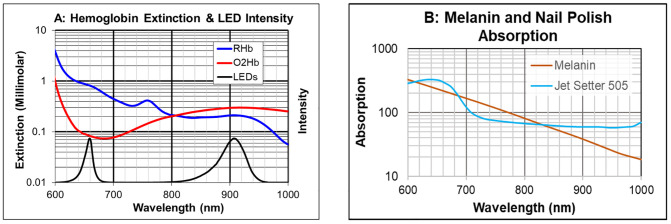
Extinction, intensity and absorption curves. A: Hemoglobin extinction curves for oxyhemoglobin (red) and reduced hemoglobin (blue). The black curve is a spectral scan of the intensity of the two LEDs from a conventional LED-based pulse oximetry sensor, also plotted as a function of wavelength. B: Absorption, on a log axis, as a function of wavelength of human melanin and of Jet Setter 505 fingernail polish [15]. (Note data for melanin absorption extracted from Fig 3 in previous reference. Data for Jet Setter 505 fingernail polish absorption was measured by authors.).

Currently available pulse oximeters use light-emitting diodes to probe the tissue in the sensor. The light that is transmitted through the tissue and received at the detector in the sensor is analyzed to calculate the arterial oxygen saturation. Considering the physics of pulse oximetry, one can see how melanin alters oxygen saturation measurements. Pulse oximetry is possible because oxyhemoglobin (O_2_Hb) and reduced hemoglobin (RHb) absorb light differently from each other, and differently at different wavelengths, as shown in the extinction curves of Fig 1A. Extinction is the opacity to light of a given substance as a function of wavelength. The higher the extinction, the greater the optical density, or opacity, of a given substance. Conversely, the lower the extinction, the more transparent the substance is. To measure arterial O_2_Hb, conventional pulse oximeters use two LEDs, one with its light output centered in the red region at approximately 660nm and a second LED centered in the near infrared region, often at approximately 900nm.

The important characteristic of LEDs, that makes pulse oximetry sensitive to melanin levels, is that they do not emit light at a single wavelength but instead output light over a range of wavelengths that can span more than 80 nanometers. The output intensity of the LEDs in a commercial pulse oximeter, as a function of wavelength, is shown in Fig 1A. Certain pigments can effectively alter the spectral content (the intensity as a function of wavelength) of these LEDs so that the ‘apparent’ LED output, and therefore the center wavelengths of these LEDs, are shifted toward the longer wavelengths. This will occur when the light absorption (another measure of the extinction) of a pigment is greater at the shorter wavelengths than it is at the longer wavelengths. This is exactly the case for melanin in the skin [15], as well as for certain brands of nail polish. The absorptions of melanin and of one particular nail polish are shown in Fig 1B.

Ideally, melanin should have no effect on pulse oximetry measurement. It is not a pigment in the arterial blood and therefore it does not pulsate with arterial pulsations. Pulse oximetry, by ‘looking’ at the pulsatile absorption of light received from the tissue-under-test, ignores the absorption of (almost) all non-pulsatile components in the tissue-under-test including venous blood, capillary blood, skin, and bone. Why then would melanin, a non-pulsatile pigment in the skin, alter measurements made by a pulse oximeter?

**Fig 2:**
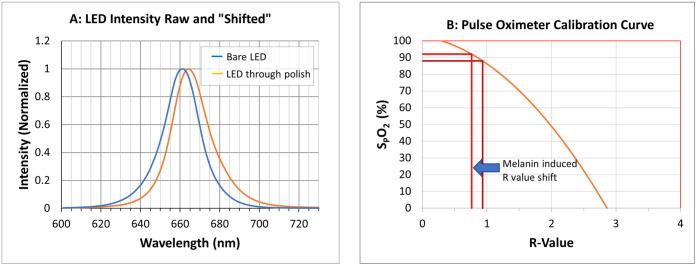
LED spectra and pulse oximeter calibration curve. A: Two spectral scans of the same 660nm LED, one without (blue) and one with (orange) “Jet Setter 505” nail polish in the optical path. The scans are scaled (each scan multiplied by a single value at all wavelengths) to make the maximum intensity of each scan equal to 1. B: A typical LED-based pulse oximeter calibration curve showing how a pigmentation shifted R-value (Ratio of 660nm/940nm extinctions) affects the measurement of oxygen saturation.

Both pigments shown in Fig 1B have greater absorption at the shorter wavelengths and lower absorption at the longer wavelengths. The impact that these pigments have on the 660nm LEDs used in pulse oximetry is to shift the spectral content, of the light out of the LED, toward the longer wavelengths as shown in Fig 2A. This figure shows a spectral scan of a 660nm LED, along with a second scan of this same LED’s light after passing through a glass slide coated with Sally Hansen, Jet Setter 505 nail polish (Coty, Inc., New York, NY. Purchased commercially for this study).

This nail polish (as with melanin) severely attenuates the shorter wavelengths of light emitted by the red LED while attenuating the longer wavelengths to a lesser extent, moving the centroid (the geometric center of mass) of this LED approximately 7nm longer in wavelength.

The effect this spectral shift has on an LED-based pulse oximeter is to alter the extinction coefficients, particularly of RHb, ‘seen’ by the pulse oximeter. This is clear by looking at the steep slope of the extinction curve for RHb at the red LED peak wavelength (~660nm) in Fig 1A. The spectral shift of this 660nm LED has a negligible effect on the O_2_Hb extinction that is read by the pulse oximeter because the extinction of O_2_Hb is much lower and changes very little in the 660nm region. A pigment (such as melanin) may cause a similar spectral shift of the 900nm LED light toward the longer wavelengths, but the extinction curves for O_2_Hb and RHb in this wavelength region are fairly flat; therefore, a shift in the centroid of the 900nm LED does not appreciably alter the extinctions that the pulse oximeter reads with the 900nm LED.

As shown in the pulse oximeter calibration curve of Fig 2B, which relates the photoplethysmographic arterial oxygen saturation values (SpO_2_) with the ratio (“R-value”) of the 660nm to the 900nm extinctions read by the pulse oximeter, the effect of the shift in the spectral content of the red (660nm) LED from the absorption by nail polish (or melanin or other pigments with a similar slope in absorption) causes an LED-based pulse oximeter to detect a lower R-value and therefore output artificially higher oxygen saturation levels, but only when the arterial oxygen saturation of the patient decreases and the RHb level increases.

Compared to the nearly 100nm spectral bandwidth (the wavelength range of the output light) of the bare red LED shown in Fig 2A, the bandwidth of a typical red single mode diode laser is less than 1nm. As a result, the sloped absorption of melanin or nail polish, as a function of wavelength, across the 1nm spectral bandwidth of a red diode laser cannot cause a shift in the spectral content of laser light and, therefore, does not alter the oxygen saturation readings made by a laser-based pulse oximeter.

We hypothesize that the effects of melanin absorption on pulse oximetry measurements are eliminated if LEDs are replaced with highly monochromatic laser-based light sources. The objectives of this research were to verify this hypothesis in an experimental benchtop study and in a clinical study.

## Materials and methods

For these studies, the lasers used in the laser-based pulse oximeters had the operational characteristics shown in Table 1.

**Table 1:** Laser characteristics.

Specification	Laser 1	Laser 2
Nominal Center Wavelength	670nm	900nm
Spectral Bandwidth (full power)	<1nm	<4nm
Laser Power	10mW	10mW

### Benchtop study

To experimentally verify this theoretically predicted behavior of LED-based and laser-based pulse oximeters under well-controlled laboratory conditions, an *in vitro* study was conducted wherein Sally Hansen Jet Setter 505 fingernail polish was used to create a spectral shift similar to that created by melanin in the skin of darkly pigmented individuals.

**Fig 3:**
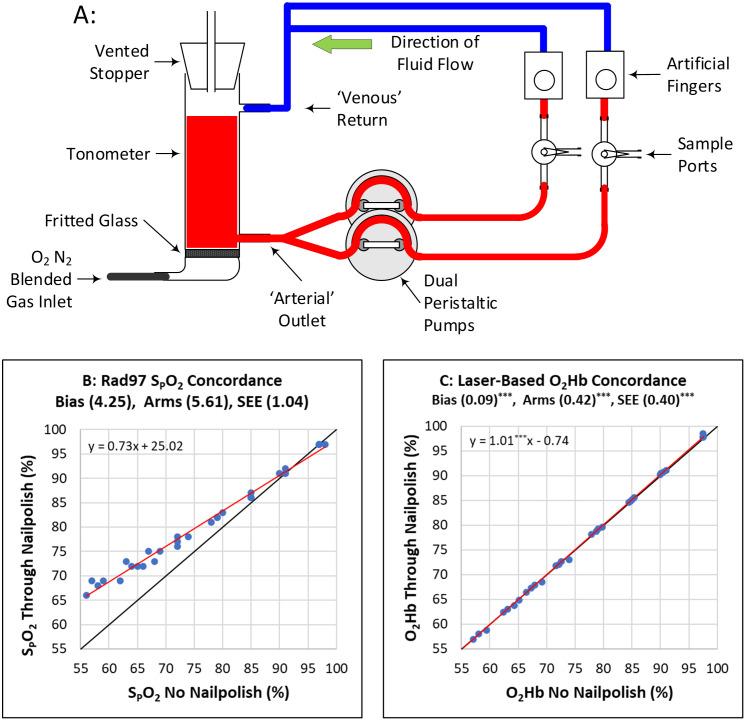
Tissue simulator and effects of nail polish on pulse oximeter measurements. A: Schematic representation of the tissue simulator system used to study the errors in pulse oximeter oxygenation measurements caused by pigmentation. B: LED-based oximeter readings (SpO_2_) with nail polish versus without nail polish in the optical path. The black 45° line is the line of identity, and the red line is the linear regression line. C: Laser-based oxygen saturation readings (O_2_Hb) with nail polish versus without nail polish in the optical path. The black 45° line is the line of identity, and the red line is the linear regression line. Note the linear regression line in this figure directly overlies the line of identity. Figs B and C include the equations for the regression lines and statistics for the Bias, Root Mean Squared error (Arms), and the Standard Error of the Estimate (SEE) as well as the equation for the regression line. Asterisks (^***^) Indicate a comparison of bias, Arms, SEE, and regression slope between data from Figs B and C that are significant to a level of p < 0.001.

Two pulse oximeters were placed on two separate artificial fingers of a human blood-based tissue simulator, shown schematically in Fig 3A. When human blood is pulsed through the circuit by a peristaltic pump the artificial fingers pulsate, and a pulse oximeter sensor placed across the artificial finger reads the oxygen saturation of the blood in the tissue simulator. Data were collected from each of the two oximeters at each of eight different blood oxygen saturation levels. At each saturation level, two data points were collected from each of the two pulse oximeters, one with a clear glass slide between the sensor and the artificial finger and a second with a nail polished glass slide replacing the clear slide.

One pulse oximeter was a conventional LED-based pulse oximeter, the Rad-97 (Masimo Corporation, Irvine CA). The second was a prototype two-wavelength laser-based pulse oximeter (developed by Zynex Monitoring Solutions, Englewood, CO). Because the spectral bandwidths of the diode lasers used in this prototype pulse oximeter are on the order of a nanometer, the centroids of the light sources in the laser-based pulse oximeter are unaltered by the optical absorption of the fingernail polish.

We hypothesized, based on the previously presented theoretical development, that the LED-based pulse oximeter would measure higher oxygen saturation values when the nail polish slide was placed in the optical path (compared to the reading received with a clear slide in the optical path) and that this effect would be particularly evident at lower oxygen saturation levels. We further hypothesized that the laser-based pulse oximeter would show no shift in oxygen saturation under the same test conditions.

### Clinical study

To confirm the theoretical prediction for more accurate laser-based photoplethysmographic measurements over LED-based measurements, regardless of pigmentation level, a clinical study on human volunteers was performed on nine darkly pigmented subjects and nine lightly pigmented subjects. To maximize the effects of the melanin on the pulse oximetry measurements, participants for this study were selected only from the top three (darkest) and bottom three (lightest) pigmentation levels of the ten-level Monk Skin Tone Scale [16]. We hypothesized that LED-based pulse oximeters would show a diverging concordance in oxygen saturation measurement between the lightly and the darkly pigmented subjects, increasing in overestimation with decreasing oxygen saturation, and that the laser-based pulse oximeter would show little to no difference as a function of pigmentation.

The study was performed using four of the prototype laser-based pulse oximeters and two LED-based pulse oximeters, a Masimo Rad-97 (same as used in the nail polish study) and a Medtronic PM1000N (Minneapolis, MN). The fingerclip sensors from all six pulse oximeters were placed on each participant so that data could be collected from all pulse oximeters simultaneously. Light blocking material was placed between the sensors to optically isolate the sensors from each other. The Rad-97 sensor was placed on the second finger of the right hand and the PM1000N sensor was placed on the second finger of the left hand, with the sensors from the laser-based pulse oximeters placed on the third and fourth fingers of each hand. Sensors for the Rad-97 and the PM1000N were placed on the participants’ fingers in accordance with the manufacturer’s instructions for use. The sensors for the prototype laser-based pulse oximeters were placed on the fingers far enough so that the fingertip rested against the fingerstop. In this position, the light path through the finger for the laser-based sensors was typically near the base of the cuticle of the fingernail. The exact light path through the finger for any of the sensors varies with finger size, fingernail size, and exact finger insertion into the sensor.

The study was performed in a manner similar to the studies required by the FDA for verification of the calibration of a pulse oximeter for marketing clearance [17]. The participants in the study had an arterial catheter placed in the radial artery and were desaturated in a stepwise manner by breathing air with controlled and monitored increasing percentages of nitrogen. At each oxygen saturation level, readings of oxygen saturation were taken from each pulse oximeter, and then an arterial blood sample was immediately withdrawn for comparison with oxygen saturation measured by a reference laboratory CO-oximeter (the ABL90 Flex Plus, Radiometer Copenhagen, Denmark). All data points where the perfusion index on the laser-based pulse oximeter was greater than 1.0% were included in the analysis.

The LED-based pulse oximeters used in this study were calibrated to functional oxygen saturation as defined in Equation 1 which is referred to as SpO_2_ on these pulse oximeters. The laser-based oximeter was calibrated to fractional oxygen saturation as defined in Equation 2, which is displayed as O_2_Hb on these laser-based pulse oximeters.


$${Functional\;Saturation\;(SpO}_{\text{2}})=\frac{{[O}_{\text{2}}Hb]}{{[O}_{\text{2}}Hb]+[RHb]}$$ (1)

$${Fractional\;Saturation\;(O}_{\text{2}}Hb)=\;\frac{\left[O_{\text{2}}Hb\right]}{\left[O_{\text{2}}Hb\right]+[RHb]+[COHb]+[metHb]}$$ (2)

Functional oxygen saturation is the percentage of hemoglobin that is bound to oxygen divided by the percentage of hemoglobin that is capable of binding to oxygen, which does not include COHb or metHb since these two forms of dyshemoglobins, or dysfunctional hemoglobins, are incapable of binding to oxygen. Fractional oxygen saturation is the percentage of the total hemoglobin that is bound to oxygen. This is arguably more important clinically as, for example, if a patient had 50% O_2_Hb and 50% COHb in their arterial blood, their functional oxygen saturation would be 100% but they would be extremely sick, whereas their fractional oxygen saturation would be 50%, clearly indicating that there is a problem. It is important to understand, however, that a pulse oximeter with only two emitters can neither measure functional nor fractional oxygen saturation in the presence of elevated levels of COHb and/or metHb [18]. These pulse oximeters are only accurate (to the measure of oxygen saturation to which they were calibrated) as long as the dyshemoglobins are at normal levels.

For the analyses in this research, readings from the LED-based pulse oximeters were compared to invasive reference functional saturation, and readings from the laser-based pulse oximeters were compared to invasive reference fractional saturation because this is how these instruments were originally calibrated.

All reference oxygen saturation levels, functional and fractional, were determined from the invasive measurements obtained from the reference CO-oximeter, which measures all four species of hemoglobin, O_2_Hb, COHb, metHb, and RHb. Note that, because these pulse oximeter calibration studies, as well as this clinical study, were performed on healthy volunteers with normal levels of COHb (~1% to 2%) and metHb (~0.7%), the difference between functional and fractional saturation measurements is small, typically less than ~3%, regardless of the reference oxygen saturation level. Nonetheless, by comparing the readings from each pulse oximeter to the invasive measurement to which it was calibrated, the difference in calibration of the devices is eliminated as a source of error from the analysis.

### Clinical study approval

This study was performed under University of California, San Francisco, Institutional Review Board approval and written informed consent was received from all participants prior to inclusion in the study. The study recruitment period was from 02-MAY-2023 to 06-JUL-2023. Because there was no assignment of participants in this clinical study to different interventions to evaluate health-related outcomes, this clinical study did not meet the requirements for registration to CinicalTrials.gov and there were no health-related outcome interventions.

### IRB information

IRB NUMBER: 21–35637

IRB APPROVAL DATE: 04/24/2023

IRB EXPIRATION DATE: 12/19/2023

Study Title: Accuracy of Pulse Oximeters with Profound Hypoxia

University of California, San Francisco, IRB

### Statistical analysis-benchtop study

A test for normality of errors from the line of identity for the two benchtop study data sets was done using the Kolmogorov-Smirnov Test of Normality. A two-sample t test was performed for comparison of means (Bias).

### Statistical analysis-clinical study

Sample size used in this study was based on sample sizes required by FDA from “Pulse Oximeters - Premarket Notification Submissions [510(k)s], Guidance for Industry and Food and Drug Administration Staff”, ISO/DIS 80601-2-61 “Particular requirements for basic safety and essential performance of pulse oximeter equipment (2017)” and the 2025 Draft version of this ISO document for pulse oximeter performance verification studies [17]. The 2017 document requires at least two, out of a minimum of ten, participants be “darkly pigmented”. The 2025 Draft version of ISO/DIS 80601-2-61 requires a minimum of six participants be in the highest three Monk Skin Tone (MST) pigmentation scale categories and six in the lowest MST categories, out of a minimum of 24 participants. Based on these references, and given that this study was specifically looking for pigmentation bias, the researchers chose to use 50% more participants than the specified minimums in the 2025 document for each of the two MST categories for a total of nine darkly and nine lightly pigmented participants.

Primary performance statistics for the concordances included: N (the number of data points in each group or subgroup), Bias (average error from the 45° line of identity), A_rms_ (the root mean squared error from the line of identity), standard error of the estimate, or SEE (the root mean squared error from the linear regression line through the data), and limits of agreement, or LOA (Bias ±1.96 times the standard deviation of the residuals in the Bias plots). A_rms_ is the primary statistic required by the FDA to quantify the accuracy and precision of a pulse oximeter because it incorporates both the measurement bias and the imprecision. The SEE is a measure of the imprecision, only. In pulse oximetry this parameter tells the scatter in the oxygen saturation measurement assuming calibration is perfect and that there is zero bias from the reference measurement. Additional analyses include comparing the variances (variance being the square of the SEEs) between the LED-based pulse oximeters and the laser-based pulse oximeters and the p-values for this comparison of variances (F-test). The F-tests were one-sided, testing whether the variances for the errors in the laser-based measurements were smaller in magnitude than the variances for the errors in the LED-based values.

## Results

### Benchtop study

As shown in Fig 3B, the LED-based pulse oximeter showed increasing divergence (falsely higher oxygen saturation readings) from the line-of-identity, at lower oxygen saturation values, when the nail polish covered slide was placed between the sensor and the artificial finger. In contrast, as shown in Fig 3C, the laser-based pulse oximeter showed no shift in oxygen saturation under identical test conditions.

### Clinical study

**Fig 4:**
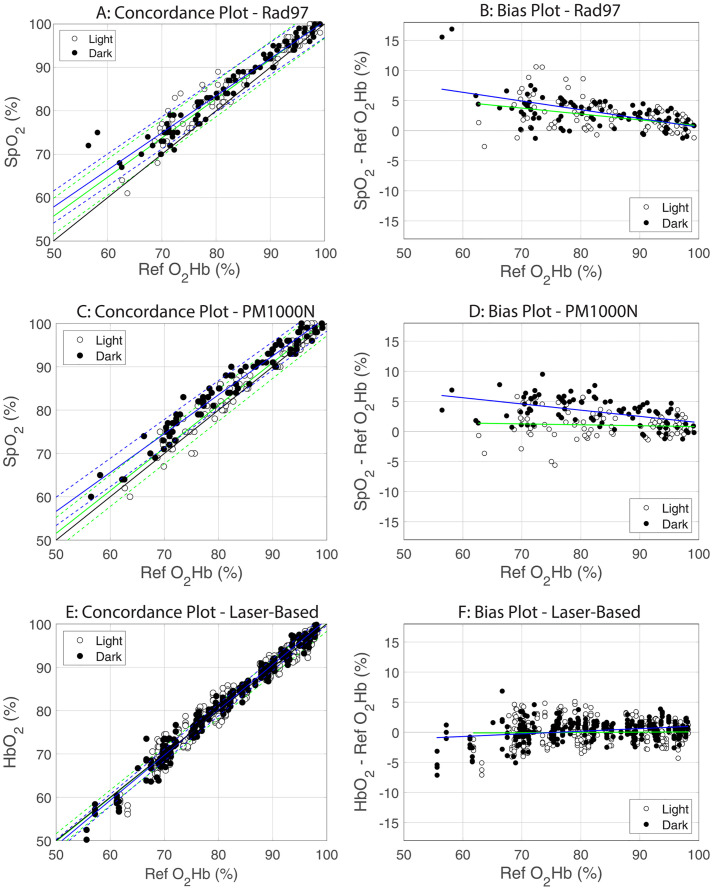
Concordance and Bias Plots for Rad-97 (plots A and B), PM1000N (plots C and D) and the Laser -Based Pulse Oximeters (plots E and F). All plots are oxygen saturation, as measured by the pulse oximeter, versus the reference oxygen saturation measurements. The invasive reference measurements for the two LED-based pulse oximeters are functional oxygen saturation values and the invasive reference measurements for the laser-based pulse oximeters are the fractional oxygen saturation values. The solid blue line in each plot is the regression line for the darkly pigmented subjects and the dashed blue lines in the concordance plots span the 95% confidence intervals on that regression. The solid green line in each plot is the regression line for the lightly pigmented subjects and the dashed green lines in the concordance plots span the 95% confidence intervals on that regression.

The results of the clinical study are shown in the Concordance plots and Bias plots in Fig 4. The plots in Figs 4A through 4D are for the LED-based pulse oximeters and the plots in Figs 4E and 4F are for the laser-based pulse oximeters. Bias plots include regression lines for the darkly and lightly pigmented participants. The blue regression line in each Bias plot is associated with the darkly pigmented participants, and the green regression line is associated with the lightly pigmented participants. The descriptive statistics for these data are shown in Table 2.

**Table 2:** Performance statistics. Descriptive statistics for all, darkly, and lightly pigmented participants. All comparisons were to invasive reference measurements. The LED-based devices (Rad-97 and PM1000N) were compared to functional saturation and the laser-based devices were compared to fractional saturation, to match how these devices were calibrated. Note, results for the laser-based devices were for the data from all four of the laser-based pulse oximeters combined.

	All Participants			Darkly Pigmented			Lightly Pigmented		
Statistic	Rad-97	PM1000N	Laser	Rad-97	PM1000N	Laser	Rad-97	PM1000N	Laser
**N**	188	188	680	93	93	317	95	95	363
**Bias (%)**	2.66	2.12	0.12	3.07	3.25	0.26	2.27	1.03	−0.01
**Arms (%)**	3.77	3.24	1.9	4.1	3.96	1.67	3.42	2.33	2.08
**SEE (%)**	2.35	2.35	1.88	2.28	2.01	1.59	2.38	2.11	2.08
**LOA (%)**	[-4.73, 10.06]	[-4.22, 8.48]	[-3.60, 3.83]	[-4.97, 11.1]	[-4.52, 11.02]	[-3.01, 3.53]	[-4.43, 8.97]	[-3.54, 5.60]	[-4.08, 4.06]

Table 3 shows the results of a comparison of variances (F-test) between the laser-based (“Laser”) pulse oximeter data and the LED-based pulse oximeters. These results show significantly greater variance of the oxygen saturation measurements made by the LED-based pulse oximeters than for the laser-based pulse oximeters for all participants combined and for the darkly pigmented subgroups at the 0.05 level.

**Table 3:** Variance statistics and comparisons. Shows variance increases from laser-based to LED-based pulse oximeters and p-values of these differences.

*Device*	*All*	*Darkly Pigmented*	*Lightly Pigmented*
Variances for each device, group, and subgroups			
Rad-97	5.48	5.14	5.63
PM1000N	5.47	4.00	4.40
Laser	3.54	2.51	4.32
Variance differences from Laser variance, for each LED device, group and subgroup			
Rad-97 – Laser	1.95 (p < 0.001)	2.63 (p < 0.001)	1.31 (p = 0.046)
PM1000N - Laser	1.94 (p < 0.001)	1.50 (p = 0.0017)	0.08 (p = 0.44)

## Discussion

As hypothesized, this clinical study showed an increasing separation in oxygen saturation measurements between LED-based pulse oximetry readings on darkly and lightly pigmented individuals (see Figs 4B and 4D)—with darkly pigmented subjects reading progressively higher as oxygen saturation decreased—and almost no difference in oxygen saturation readings as a function of pigmentation when measurements were made on a laser-based pulse oximeter (see Fig 4F). (The prototype laser-based pulse oximeter device discussed in this manuscript is for investigational use only and not yet approved for clinical use by the FDA.) These results fully support the previously presented theoretical analysis and the benchtop study results.

The laser-based readings plotted in Fig 2F show a slight reverse bias of oxygen saturation differences from the bias demonstrated by the LED-based pulse oximeters, in that, as the arterial oxygen saturation decreased, the lightly pigmented participants read slightly higher than the darkly pigmented participants. This may simply be a function of random variability, or it could be due to lower received light levels at the photodetector (in the sensor) due to the high optical absorption of melanin in these participants, which results in a lower signal-to-noise ratio compared to readings on lightly pigmented individuals.

These clinical results were predicted theoretically and demonstrated experimentally in the nail polish study presented above. Looking at the variances measured only for the lightly pigmented participants (Table 3), the PM1000N LED-based pulse oximeter did not demonstrate significantly greater variability than the laser-based pulse oximeter. This is not surprising, given that many commercially available pulse oximeters were primarily calibrated on lightly pigmented volunteers and that pigmentation levels in lightly pigmented persons do not vary as greatly as pigmentation levels in more darkly pigmented persons, causing less variability in the measurement error in these individuals.

The increased scatter in LED-based pulse oximeter readings with measurements on individuals with a mixture of different pigmentation levels has been noted by a number of researchers. As early as 2005, researchers showed that three different LED-based pulse oximeters demonstrated an increasing difference (error) in arterial oxygen saturation between darkly and lightly pigmented study participants, with the darkly pigmented participants reading progressively higher as arterial oxygen saturation decreased [3]. This concerning behavior of LED-based pulse oximeters was brought back to light in a 2020 article which gained a great deal of press and sparked a flurry of follow on studies into pigmentation bias in pulse oximetry [4]. In this study, the researchers found that black patients were more than three times more likely to have a pulse oximetry reading equal to or greater than 92% when their invasive oxygen saturation measurement was less than 88% compared to white patients.

Another group of researchers performed a retrospective analysis of paired SpO_2_ and arterial blood gas values for oxygen saturation from over 87,000 patients who self-identified as black, Asian, Hispanic or white [19]. This 2021 report found that hidden hypoxia (defined in this study as paired measurements where SpO_2_ ≥ 88% when invasive arterial oxygen saturation < 88%) was more prevalent, the variability in the SpO_2_ error was greater, and the in-hospital mortality was higher, in black patients than in white patients. Another study paired SpO_2_ and invasive arterial oxygen saturation measurements, taken within one minute of each other, in 294 preterm infant patients identified as black (42%) or white (58%) on their birth certificates [20]. In this 2022 report, the research found greater variability in measurement error, positive measurement bias, and occult hypoxemia (defined as SpO_2_ ≥ 90% when invasive arterial oxygen saturation <85%), for the black infants compared to the white infants. In a 2024 report, researchers performed a controlled desaturation study on 146 healthy participants, including 25 lightly pigmented participants (Class I and II on the Fitzpatrick skin color scale) and 43 darkly pigmented participants (Class V and VI on the Fitzpatrick skin color scale) [6]. In this study, participants had a radial artery catheter in place, and a stepwise desaturation was performed from 60% to 100%, with paired arterial oxygen saturation and SpO_2_ readings taken each step once a stable oxygen saturation level was obtained. One significant difference in this study was that the authors also looked at the contribution of low perfusion at the pulse oximeter sensor site to SpO_2_ measurement error. They found increased pigmentation levels were associated with increased positive measurement error (falsely elevated SpO_2_ levels), and that low perfusion appeared to further exacerbate this effect. This study defined occult hypoxia as arterial oxygen saturation <88% when SpO_2_ was between 92 and 96%, and found that the incidence of occult hypoxia was, once again, greater in the more darkly pigmented participants. Similar results were reported in 2024 in a prospective study on 57 black and 56 white critically ill patients [21]. The study found a correlation between the magnitude of the error bias and skin tone, increasing overestimating SpO_2_ as a function of increasingly dark pigmentation of the patient.

Finally, a 2025 study collected data on 320 children in hospital for cardiac catheterization [22]. These researchers recorded SpO_2_ from two different manufacturers’ LED-based pulse oximeters at the same time that arterial blood was drawn for measurement by CO-oximetry. They also found that the positive error in SpO_2_, along with the imprecision and the frequency of occult hypoxia, was greater in children with darker skin compared to those with lighter skin.

The findings of all the studies described above were in line with what was found in this study for the LED-based pulse oximeters. The difference with the laser-based pulse oximeter tested in this study was that the pigmentation bias was no longer evident. This is further substantiated by looking at the 95% confidence intervals in the three concordance plots of Fig 4, where the line of identity is completely enclosed within the confidence intervals only for the laser-based pulse oximeters (Fig 4E), despite the wider confidence intervals seen in the LED-based pulse oximeter concordance plots due to the increased variability associated with the LED-based measurements.

While it would be convenient if the effects of melanin on pulse oximetry in a clinical study were as clear and binary as the effects of nail polish *in vitro*, as shown in Fig 3, unfortunately, there is a great deal more variability when testing the effects of melanin in humans. A large part of this variability is due to the natural variability in the depth and concentration of melanin of participants at the fingertip, even when selecting only the most and least pigmented participants.

Additionally, pulse oximeter sensors position the light sources and photodiodes of the sensors such that light is projected through the finger near the base of the cuticle. This means that if the finger in the sensor is small, the light path through the tissue may be just proximal to the cuticle. And, if the finger is larger, or perhaps the fingernail is longer, the light path may be distal to the cuticle, through the nail. In darkly pigmented individuals it is common for the finger to be much darker proximal to the cuticle and considerably lighter distal to the cuticle. Thus, the exact positioning of the sensor on the finger, the design of the sensor with respect to where it is intended to position the light sources on the finger, as well as the finger size, can cause a great deal of variability in how much melanin a sensor sees as the light passes through the tissue. Therefore, one can expect any clinical study of pulse oximetry, such as the one presented here, to see a great deal of variability in the effects of melanin on the oxygen saturation readings of LED-based pulse oximeters [3].

There are limitations in this clinical study. This clinical study had a small sample size; only nine darkly and nine lightly pigmented participants were included in this study. A second limitation, as described above, is the inability to ensure that the participants in each of the two subgroups, specifically the lightly and darkly pigmented subgroups, had the same concentration and path length of melanin in the optical path of the pulse oximeter sensors in use. Finally, four of the five authors of this study were employees of Zynex Monitoring Solutions, that owns the laser-based pulse oximetry prototypes used in these studies and therefore have a conflict of interest and may be biased in this work.

## Conclusions

Given the theoretical development presented here, in combination with the empirical evidence from the nail polish study and the clinical study, we conclude that conventional LED-based pulse oximeters will, on average, read higher on darkly pigmented persons than on lightly pigmented persons, at the lower oxygen saturation values. Additionally, the lack of pigmentation bias seen in the results from the laser-based pulse oximeters further confirms that the problem of pigmentation bias arises from the wide spectral bandwidth of the red LEDs used in conventional pulse oximetry.

Substituting laser light sources for LED light sources in commercial pulse oximeters could eliminate pigmentation bias in pulse oximetry monitoring and oxygen administration. Eliminating pigmentation bias would eliminate the increased frequency of adverse clinical outcomes in darkly pigmented patients who need supplemental oxygen compared to those with lighter pigmentation levels.
